# Editorial: Combining a non-invasive transcranial stimulation technique with another therapeutic approach: mechanisms of action, therapeutic interest and tolerance

**DOI:** 10.3389/fneur.2024.1440696

**Published:** 2024-07-16

**Authors:** Jean-Pascal Lefaucheur, Jean-Paul Nguyen, Helena Knotkova, Simone Rossi, Julien Nizard

**Affiliations:** ^1^UR 4391, Excitabilité Nerveuse et Thérapeutique, Université Paris-Est Créteil, Créteil, France; ^2^Unité de Neurophysiologie Clinique, Hôpital Henri Mondor, Assistance Publique - Hôpitaux de Paris (AP-HP), Créteil, France; ^3^Unité de stimulation transcrânienne, clinique Bretéché, groupe Elsan, Nantes, France; ^4^MJHS Institute for Innovation in Palliative Care, New York, NY, United States; ^5^Department of Family and Social Medicine, Albert Einstein College of Medicine, Bronx, NY, United States; ^6^Siena Brain Investigation and Neuromodulation Lab (Si-BIN Lab), Department of Medicine, Surgery and Neuroscience, Neurology and Clinical Neurophysiology Section, University of Siena, Siena, Italy; ^7^Service Douleur, Soins palliatifs et de Support, Hôpital Laennec, Centre Hospitalier Universitaire de Nantes, Nantes, France; ^8^UMR 1229, Médecine Régénératrice et Squelette, INSERM, Université de Nantes Université, Nantes, France

**Keywords:** repetitive transcranial magnetic stimulation (rTMS), transcranial direct current stimulation (tDCS), transauricular vagus nerve stimulation (taVNS), non-invasive neuromodulation techniques, combined strategy

Numerous publications have attested to the therapeutic efficacy of non-invasive neuromodulation techniques, such as repetitive transcranial magnetic stimulation (rTMS) and low-intensity transcranial electrical stimulation (tES), but also peripheral magnetic (pMS) or electrical (pES) stimulation techniques, particularly applied to certain cranial nerves, such as transauricular vagus nerve stimulation (taVNS) or occipital nerve stimulation (ONS). These nerves can also be stimulated invasively (iVNS, iONS) using surgically implanted electrodes and pulse generators. These methods have been used in the treatment of various neurological conditions, such as chronic pain, cognitive disorders, poststroke rehabilitation, or movement disorders. In these different domains, evidence suggests that therapeutic efficacy could be improved by combining neuromodulation techniques with other types of non-pharmacological approaches, such as motor or cognitive tasks or training. In this Research Topic collection, we gathered together nine publications evaluating such a combined strategy in different clinical contexts. They concerned the treatment of various pain conditions (Wandrey et al., Agostinho et al.), even associated with cognitive impairment (Caloc'h et al.), a pure cognitive disorder (Horczak et al.), disorder of consciousness (Zhuang et al.), motor stroke rehabilitation (Wang et al., Qi et al.), dystonia (Bleton et al.), or motor, language, or cognitive enhancement before brain surgery (Boccuni et al.). Concerning the type of neuromodulation technique, publications have addressed the value of tDCS (Wandrey et al., Agostinho et al., Horczak et al., Bleton et al.), tDCS or rTMS (Boccuni et al.), tDCS and taVNS (Zhuang et al.), taVNS or iVNS (Wang et al.), rTMS and iONS (Caloc'h et al.), and tES, rTMS, pES, or pMS (Qi et al.). Complementary techniques were cognitive training (Caloc'h et al.), mirror therapy or behavioral interventions (Agostinho et al., Horczak et al., Qi et al., Boccuni et al.), motor training or rehabilitation (Wang et al., Bleton et al.), local anesthetic infiltrations (Wandrey et al.), or just a combination of two neuromodulation techniques (Zhuang et al.).

First, Wandrey et al. show in a randomized sham-controlled trial that anodal or cathodal tDCS delivered to the primary motor cortex (M1) did not significantly enhance pain alleviation provided by subsequent local anesthetic infiltrations (primarily targeting the sphenopalatine ganglion) compared to sham tDCS in patients with either trigeminal neuralgia or persistent idiopathic facial pain. However, due to a high dropout rate, only a few patients completed the study (six, three, and four patients for anodal, cathodal, and sham tDCS, respectively), which therefore remains inconclusive and warrants further investigation in larger series.

Second, Agostinho et al. review the literature on the value of combining tDCS with other non-pharmacological approaches in the field of pain. These authors specifically highlight their own experience with combining anodal tDCS of M1 and mirror therapy to treat phantom limb pain. They showed that applying this therapeutic strategy at an early stage from the onset of symptoms produced impressive pain relief with long-lasting after-effects. In this perspective article, the authors recommend applying such an intervention at the acute stage of a painful disease, or as early as possible to limit maladaptive plasticity and prevent the chronification of a pain syndrome.

Third, Caloc'h et al. address a clinical condition combining pain and cognitive impairment, secondary to traumatic brain injury. In the reported case, the patient was first treated with bilaterally iONS to relieve chronic refractory headaches (8 years after the head trauma). Two years later, he was treated with a 6-week protocol combining rTMS delivered to multiple cortical sites and cognitive training (CogT) targeting memory, language, and visuospatial functions. Pain relief and cognitive improvement were observed after iONS but the multisite rTMS-CogT protocol provided additional significant improvement on apathy, depression, and anxiety.

Fourth, Horczak et al. show in a parallel randomized sham-controlled study involving 17 participants that active anodal tDCS of the left dorsolateral prefrontal cortex performed prior to sessions of cognitive behavioral therapy (attention task) did not provide significant additional improvement over sham stimulation in treating rumination linked to negative mood. Again, a too small sample size possibly prevented statistical differences between active and sham tDCS-combined protocols from being achieved.

Fifth, Zhuang et al. describe a protocol for a randomized sham-controlled study of the combination of tDCS and taVNS to treat disorders of consciousness. The goal of such a strategy is to enhance bottom-up thalamo-cortical connections using bilateral taVNS and simultaneously increase top-down cortico-cortical connections using high-definition tDCS (HD-tDCS) centered on Pz with four return electrodes placed at Cz, P3, P4, and POz to target the precuneus and the posterior parietal cortex. All patients will undergo a 4-week treatment and will be evaluated on clinical aspects and electroencephalogram (EEG) microstates.

Sixth, Wang et al. report a systematic review of the literature (with meta-analysis) on the efficacy of taVNS or iVNS combined with motor training in the rehabilitation of poststroke upper limb motor dysfunction. Ten trials with 335 patients were included in the meta-analysis. Regarding upper extremity motor function, based on Fugl-Meyer assessment scores, VNS combined with other treatment options had immediate and long-term (1–3 months) beneficial effects compared to that of the control treatment. Subgroup analyses showed that taVNS may be superior to iVNS, that a stimulation frequency set at 20 Hz may be superior to higher frequencies, and that VNS combined with integrated treatment may be superior to VNS combined with upper extremity training alone. Beyond motor improvement, VNS may improve activities of daily living and depression, but perhaps not the overall quality of life. The mechanisms underlying the effects of VNS on motor recovery in stroke patients remain unclear, potentially related to a non-specific modulation of cortical network excitability, which is able to facilitate functional recovery specifically related to the task performed in combination.

Seventh, Qi et al. review the literature on the potential benefits of combining various non-invasive stimulation techniques (tES, rTMS, pES, pMS) with action observation training in poststroke rehabilitation. Furthermore, they discussed how tES or rTMS over the contralesional hemisphere or the lesioned hemisphere combined with pES or pMS of the paretic limbs during motor observation followed by action execution have super-additive effects to potentiate the effect of conventional rehabilitation strategies.

Eight, Bleton et al. report a case series of five patients with cervical dystonia poorly controlled by botulinum toxin injections and treated by repeated daily sessions of anodal tDCS of the cerebellum combined with oriented motor training, specifically developed to treat this clinical condition. The combined strategy produced a more striking and prolonged improvement in dystonia and dystonia-related pain than the application of cerebellar tDCS alone.

Ninth, Boccuni et al. describe a study protocol to assess the value of 10–20 sessions (one or two sessions each weekday, 30-min duration) of “inhibitory” non-invasive brain stimulation (NIBS) protocol (either low-frequency rTMS or mostly cathodal tDCS) coupled with intensive motor, language, or cognitive training session (30-min duration) in a series of patients with brain tumor before the surgical removal. The objective of this protocol is to reduce the activation of the brain regions concerned by the surgery by locally applying inhibitory neuromodulation and to concomitantly promote neuroplasticity and increase the activation of alternative brain pathways by intensive training with specifically adapted tasks. Thus, the goal of this strategy called “neuromodulation-induced cortical prehabilitation” (NICP) is to reduce the functional relevance of cortical areas by applying inhibitory NIBS and then to facilitate their resection with reduced risks of neurological sequelae. The post-surgical assessment will be based on clinical outcomes (motor function, balance, cognitive and language performance, quality of life), as well as on functional neuroimaging and navigated TMS mapping.

This Research Topic collection clearly shows that the non-invasive neuromodulation procedures (tDCS, rTMS, taVNS) that can be used in combination with other nonpharmacological approaches are extremely varied, as are the potential therapeutic indications for these combinations. These publications, although innovative, have significant limitations. There were only two randomized sham-controlled studies and both report negative results of a tDCS protocol (Wandrey et al., Horczak et al.). This may be explained by a small total sample size (13–17 patients), further divided into parallel groups. Another possible explanation is the fact that the complementary therapeutic intervention (local anesthetic infiltration or cognitive behavioral therapy) led to a “ceiling” effect which did not allow to highlight an additional effect of tDCS. In contrast beneficial effects of NIBS procedures (rTMS or tDCS) were reported in two other studies (Caloc'h et al., Bleton et al.), but based on open-labeled single or few case reports. The other articles are points of view, literature reviews or meta-analyses (Agostinho et al., Wang et al., Qi et al.) or study protocol description (Zhuang et al., Boccuni et al.).

In terms of neuromodulation techniques to be used in a combined strategy, there is a preference for tES or taVNS, which can be applied more easily than rTMS, including at home. Invasive procedures, such as iVNS and iONS, were also addressed in the present studies and should not be neglected to promote long-term benefits in clinical practice. An additional interesting point is also the timing of the intervention, an early application being particularly promising as suggested by Agostinho et al..

The efficacy of non-invasive neuromodulation techniques could be increased in the future by additional improvements, such as a better definition of indications, the personalization of targeting (including new targets), or the optimization of maintenance protocols. The simplification of procedures, the portability of the devices, and their lower costs will also contribute to their diffusion among patients.

Such combined protocols in any case present a good safety profile, but they need to be better standardized and better evaluated, as the available scientific data still remains largely insufficient. The publications of this Research Topic collection do not yet make it possible the establishment of good practice recommendations regarding any of these combined therapeutic approaches. This of course underlines the importance of new controlled studies on larger sample sizes to confirm the potential benefits of such treatment combinations in the different indications described in this Research Topic, but also very probably in many others which will emerge in the near future.

## Author contributions

J-PL: Writing – original draft, Writing – review & editing. J-PN: Writing – review & editing. HK: Writing – review & editing. SR: Writing – review & editing. JN: Writing – review & editing.

